# Transglutaminase-mediated glycosylation improves the physicochemical properties and in vitro hypolipidemic activity of oyster peptides

**DOI:** 10.1016/j.fochx.2026.104027

**Published:** 2026-05-23

**Authors:** Qianqian Huang, Zhongqin Chen, Mingtang Tan, Huina Zheng, Haisheng Lin, Jialong Gao, Xiaoming Qin, Wenhong Cao

**Affiliations:** a College of Food Science and Technology, Guangdong Ocean University, Zhanjiang 524088, China; b National Research and Development Branch Center for Shellfish Processing, Zhanjiang 524088, China; c Guangdong Provincial Key Laboratory of Aquatic Products Processing and Safety, Zhanjiang 524088, China

**Keywords:** Oyster peptide, Glycosylation, Transglutaminase, Glycopeptide, Physicochemical properties, Hypolipidemic activity, Digestive stability

## Abstract

Oyster peptides exhibit diverse bioactivities but suffer from poor stability and digestive degradation, limiting functional food applications. To overcome these, this study employed transglutaminase (TGase)-catalyzed glycosylation to conjugate glucosamine (GlcN) with an oyster peptide ultrafiltration fraction (UF3), and evaluated the structural, physicochemical, and hypolipidemic properties. Results showed that glycosylation induced a β-sheet to α-helix shift and transformed the microstructure into a smooth, thin-film network. These changes reduced average particle size, lowered surface hydrophobicity, and enhanced solubility and thermal stability. The glycosylated product (GUF3) exhibited improved inhibitory activities against pancreatic lipase and cholesterol esterase. Moreover, GUF3 showed superior gastrointestinal resistance, retaining higher enzyme inhibition after intestinal digestion than UF3. In free fatty acids-induced HepG2 cells, GUF3 more effectively reduced lipid accumulation, total cholesterol, and triglycerides. Collectively, TGase-catalyzed glycosylation improves the structure, digestive stability, and hypolipidemic activity of oyster peptides, supporting GUF3 as a promising ingredient for hyperlipidemia management.

## Highlights

• TGase-catalyzed GlcN glycosylation modified oyster peptide UF3 to GUF3.
• Glycosylation shifted secondary structure to α-helix and formed a smooth network.
• GUF3 showed enhanced solubility, thermal stability, and digestive resistance.
• GUF3 exhibited superior in vitro hypolipidemic activity in enzyme and cell assays.

## Graphical abstract

**Figure:**
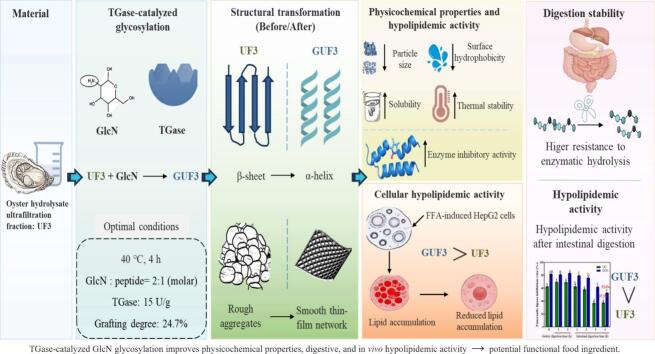

## 1 Introduction

Oyster, a nutrient-rich marine bivalve, is abundant in bioactive components including proteins, polysaccharides and trace elements, and has been widely explored for applications in food, nutraceutical and pharmaceutical industries (Du et al., 2024). Oyster peptides (OPs), small molecular fragments derived from oyster protein hydrolysis, have attracted extensive research attention due to their diverse biological activities, such as antioxidant, antimicrobial, immunomodulatory and hypolipidemic effects (Ma et al., 2022). Among these properties, the hypolipidemic activity of OPs makes it a promising natural functional factor for the prevention and amelioration of lipid metabolic disorders, which has great potential in the development of hypolipidemic functional foods. However, the practical application of OPs is severely limited by their poor stability, especially their high susceptibility to enzymatic hydrolysis during gastrointestinal (GI) digestion, which leads to significant loss of bioactivity (Zhao et al., 2024). For instance, previous studies have reported that the antioxidant activity of oyster protein hydrolysates decreased markedly after GI digestion and short-term storage (Xu et al., 2021). Thus, developing effective modification strategies to improve the digestive stability and retain the bioactivity of OPs has become a research priority in food science.

Glycosylation, a typical biochemical modification method, has emerged as an effective approach to improve the physicochemical properties and bioactivity stability of proteins/peptides in recent years (Huang et al., 2025a). This technology achieves structural modification by covalently linking sugar molecules to the amino acid residues of peptides, thereby regulating their spatial conformation, physicochemical properties, and biological activity (Zhao et al., 2023). Glycosylated peptides have been proven to exhibit enhanced physicochemical stability, digestive resistance and bioactivity compared with their unmodified counterparts (Moradi et al., 2016). Currently, glycosylation is mainly realized through two approaches: the Maillard reaction (non-enzymatic glycation) and enzyme-catalyzed glycosylation. Although the Maillard reaction is a simple method for preparing glycopeptide (GP) conjugates, it requires harsh reaction conditions (high temperature and long reaction time) and inevitably generates harmful by-products such as advanced glycation end products (AGEs), which pose potential health risks and limit its application in food processing (Gottardi et al., 2014).

In contrast, transglutaminase (TGase)-catalyzed enzymatic glycosylation proceeds under mild conditions (typically 37–40 °C) and avoids the production of toxic by-products, making it a safer and more food-friendly modification strategy (Liu et al., 2022). TGase was originally found to catalyze protein cross-linking by forming isopeptide bonds between the γ-carboxamide group of glutamine (Gln) residues and the ε-amino group of lysine residues (Zhang et al., 2022). Beyond this cross-linking function, TGase also catalyzes the formation of isopeptide bonds between the free amino group of amino sugar molecules and the γ-carboxamide group of Gln residues on peptide chains, achieving efficient and specific conjugation of sugars and peptides while well preserving the native bioactive conformation of peptides (Wang et al., 2024; Yu et al., 2025). Previous studies have demonstrated that TGase-mediated glycosylation can significantly improve the thermal stability, antioxidant activity and functional properties of various food-derived peptides, such as fish skin gelatin hydrolysate and casein hydrolysate (Hong et al., 2014; Wang et al., 2025).

Glucosamine hydrochloride (GlcN), a natural amino monosaccharide with reactive amino and hydroxyl groups, is an ideal glycosyl donor for TGase-catalyzed glycosylation. Besides serving as a modification substrate, GlcN also possesses inherent physiological activities, including anti-inflammatory, antioxidant and hypolipidemic effects, which can modulate hepatic lipid metabolism and alleviate non-alcoholic fatty liver disease induced by a high-fat diet (Lai et al., 2024; Li et al., 2023; Ryu et al., 2025). The covalent conjugation of GlcN with bioactive peptides is expected to achieve a synergistic enhancement of bioactivity, in addition to improving the structural stability of peptides.

GlcN has been successfully applied in the glycosylation modification of fish gelatin peptides, gluten peptides and zein peptides, and the modified products showed significant improvements in physicochemical stability and bioactivity (Gottardi et al., 2014; Hong et al., 2014; Liu et al., 2022). However, systematic research on TGase-catalyzed GlcN glycosylation of OPs remains relatively limited, and there is a lack of in-depth investigations on the effects of this modification on the structural characteristics, digestive stability and in vitro hypolipidemic activity of OPs. In addition, most existing studies on OPs focus on ungraded hydrolysates, while the modification and bioactivity of small molecular weight oyster hydrolysate ultrafiltration fractions have not been fully explored.

Based on the above research background, this study prepared glucosamine-oyster peptide conjugates (GUF3) via TGase-catalyzed GlcN glycosylation using the oyster hydrolysate ultrafiltration fraction UF3 (UF3, <3 kDa) as the raw material. The structural changes of UF3 before and after glycosylation were systematically characterized by multiple analytical techniques. The effects of glycosylation on the physicochemical properties (particle size, zeta potential, thermal stability, surface hydrophobicity and solubility) and digestive stability of UF3 were also investigated. Furthermore, the in vitro hypolipidemic activity of GUF3 was evaluated by pancreatic lipase (PL) and cholesterol esterase (CE) inhibition assays, and further verified using a free fatty acid (FFA)-induced HepG2 cell model. This study aims to elucidate the relationship between TGase-catalyzed GlcN glycosylation and the hypolipidemic activity of UF3, provide a mild and efficient modification strategy for improving the digestive stability and bioactivity of OPs, and lay a theoretical foundation for the high-value utilization of oyster resources and the development of natural hypolipidemic functional foods.

## 2 Materials and methods

### 2.1 Materials

Oysters (*Crassostrea hongkongensis*) were purchased from Guangdong Yuezhibao Aquatic Products Co., Ltd. (Zhanjiang, China). Shengmeinuo oyster peptides (SOP) were obtained from Hainan Shengmeinuo Biotechnology Co., Ltd., Chengmai Branch (Chengmai, China). Neutral protease (100 U/mg), complex protease (120 U/mg), GlcN, TGase (50 U/g), PL (35 U/mg), Orlistat, o-phthaldialdehyde (OPA), sodium dodecyl sulfate (SDS), artificial gastric juice, and artificial intestinal juice were all supplied by Shanghai Yuanye Bio-Technology Co., Ltd. (Shanghai, China). *p*-Nitrophenyl palmitate (PNPP), 4-nitrophenyl butyrate (p-NPB), CE (>15 U/mg), β-mercaptoethanol, sodium tetraborate, and potassium bromide were obtained from Shanghai Macklin Biochemical Technology Co., Ltd. Fatty acid-free bovine serum albumin was purchased from Meliun Biotechnology Co., Ltd. Sodium palmitate and sodium oleate were obtained from Beijing Solarbio Science & Technology Co., Ltd. All other chemicals used in this study were of analytical grade.

### 2.2 Preparation of oyster enzymatic hydrolysate and ultrafiltration fractions

The preparation of oyster enzymatic hydrolysate and its ultrafiltration fractions followed the method previously established by our research group (Zhu et al., 2021). Briefly, fresh oyster meat was washed, drained, homogenized with distilled water (1:3, g/mL), and adjusted to pH 7.0 with 0.2 mol/L NaOH. Hydrolysis was performed at 50 °C for 4 h using neutral or complex protease at an enzyme-to-substrate ratio of 1000:1 (U/g). After heating in boiling water for 10 min to inactivate enzymes, the mixture was centrifuged (4 °C, 12,000 rpm, 20 min). The supernatant was concentrated, freeze-dried, and stored at −20 °C.

The oyster neutral protease hydrolysate (ONPH) was sequentially fractionated with ultrafiltration membranes (8, 5, and 3 kDa). Fractions of 8–5 kDa (UF1), 5–3 kDa (UF2), and < 3 kDa (UF3) were collected, freeze-dried, and stored at −20 °C for further use.

### 2.3 Preparation conditions for the glycosylated product

Based on the method of Liu et al. (2022) with modifications and our preliminary single-factor experimental results **(Supplementary Fig. S2)**, precisely weighed UF3 powder was dissolved in distilled water, mixed with GlcN, and the pH was adjusted to 7.0. TGase (15 U/g) was then added to initiate the reaction at 40 °C for 4 h, with a GlcN-to-UF3 molar ratio of 2:1. Unreacted GlcN (215.6 Da) was remo*v*ed by dialysis using a dialysis membrane with a 300 Da molecular weight cut-off for 48 h. The retentate was collected, freeze-dried, and stored at −20 °C for later use. The glycosylated product was named GUF3.

### 2.4 Degree of grafting

The degree of grafting (DG) of the glycosylated product was determined using the OPA method (Zhang et al., 2021). Briefly, 4 mL of OPA reagent was mixed with 200 μL of diluted sample for 1 min, incubated at 35 °C for 2 min in the dark, and the absorbance was measured at 340 nm. The OPA reagent was prepared by dissolving 80 mg OPA in 2 mL methanol, followed by sequential addition of 50 mL sodium tetraborate buffer (0.1 mol/L), 5 mL SDS (20%, *w*/*v*), and 200 μL β-mercaptoethanol. The mixture was diluted to 100 mL with ultrapure water. DG was calculated using Formula (2-1):

(2-1) $$\mathrm{DG}\;\left(\%\right)=\frac{A_{0}-A_{1}}{A_{0}}\times 100\%$$

where *A*₀ and *A*₁ represent the absorbance before (sugar-peptide mixture) and after glycosylation reaction, respectively.

### 2.5 Determination of in vitro enzyme inhibitory activity

Pancreatic lipase inhibitory (PLI) activity and cholesterol esterase inhibitory (CEI) were assessed following Shen et al. (2023) with minor modifications. For PLI, PL was dissolved in Tris-HCl buffer (50 mmol/L, pH 8.0), centrifuged (5000 rpm, 10 min), and diluted to 0.35 U/mL. PNPP substrate (1 mg/mL) was prepared in isopropanol and diluted with Tris-HCl buffer containing 0.1% gum arabic and 0.2% sodium deoxycholate. Briefly, 50 μL of sample and 50 μL of PL solution were mixed in a 96-well plate and incubated at 37 °C for 10 min. Then, 50 μL of PNPP solution was added and incubated at 37 °C for 20 min in the dark. Absorbance was measured at 405 nm using a microplate reader. The inhibition rate was calculated using Formula (2-2).

(2-2) $$\text{Inhibition rate of}\;\mathrm{PL}\;\left(\%\right)=\left[1-\left(\frac{A_{\mathrm{sample}}-A_{\mathrm{blank}}}{A_{\mathrm{test}}-A_{\mathrm{control}}}\right)\right]\times 100\%$$

In the formula: *A*_sample_, *A*_blank_, *A*_test_, and *A*_control_ represent the absorbance of reaction systems containing the following components: sample + PL + PNPP, sample + buffer + PNPP, distilled water + PL + PNPP, and distilled water + buffer + PNPP, respectively.

For CEI, p-NPB substrate (2 mg/mL) was prepared in potassium phosphate buffer (0.1 mol/L, pH 7.0) containing 0.1 M NaCl and 0.05 M sodium taurocholate. Sample (50 μL) and p-NPB (50 μL) were mixed in a 96-well plate and incubated at 37 °C for 10 min. CE solution (50 μL, 0.255 U/mL) was then added and incubated at 37 °C for 20 min in the dark. Absorbance was measured at 405 nm, and inhibition rate was calculated using Formula (2-3).

(2-3) $$\text{Inhibition rate of}\;\mathrm{CE}\;\left(\%\right)=\left[1-\left(\frac{A_{\mathrm{sample}}-A_{\mathrm{blank}}}{A_{\mathrm{test}}-A_{\mathrm{control}}}\right)\right]\times 100\%$$

In the formula: *A*_sample_, *A*_blank_, *A*_test_, and *A*_control_ represent the absorbance of reaction systems containing the following components: sample + CE + p-NPB, sample + buffer + p-NPB, distilled water + CE + p-NPB, and distilled water + buffer + p-NPB, respecti*v*ely.

### 2.6 Structural characterization of glycosylated products

#### 2.6.1 Molecular weight determination

Molecular weight changes of UF3 before and after glycosylation were analyzed by high-performance liquid chromatography (HPLC) (Waters 2695, USA) using a TSKgel G2000 SWXL column (300 mm × 7.8 mm) at 30 °C. The mobile phase was acetonitrile-water-trifluoroacetic acid (40:60:0.1, *v*/v) at 0.5 mL/min, with detection at 220 nm. The molecular weight calibration curve was established using the following standards: cytochrome C (Mw 12,384), aprotinin (Mw 6500), bacitracin (Mw 1422), Gly-Gly-Tyr-Arg (Mw 451), and Gly-Gly-Gly (Mw 189).

#### 2.6.2 Intrinsic fluorescence spectroscopy

UF3, and GUF3 were dissolved in ultrapure water (0.1 mg/mL). Fluorescence spectra were recorded using an RF-5301PC spectrofluorophotometer (Shimadzu, Japan). Excitation was set at 280 nm, and emission was scanned from 310 to 420 nm (slit width: 5 nm) (Zhao et al., 2024). All measurements were conducted in triplicate.

#### 2.6.3 UV–Vis spectra measurement

The sample was prepared with ultrapure water to a concentration of 0.1 mg/mL, and its absorbance was subsequently scanned from 190 to 400 nm using a Cary 60 UV–Vis spectrophotometer (Agilent Technologies, USA).

#### 2.6.4 Fourier transform infrared spectroscopy measurement

Following the method of Wang et al. (2022), the sample was thoroughly mixed with dried KBr at a ratio of 1:100, ground, and pressed into a transparent pellet using a hydraulic press. The pellet was then analyzed by FT-IR (TENSOR 27, Bruker, Germany) with a scanning range of 4000–400 cm^−1^, a resolution of 4 cm^−1^, and 32 scans. Background correction was performed using a blank KBr pellet.

#### 2.6.5 Scanning electron microscopy

The dried powder samples were fixed on double-sided adhesive tape and sputter-coated with a gold layer under vacuum (Wang et al., 2022). The microstructure of the samples was observed using a scanning electron microscope (SEM) (JSM-7610F, Tokyo, Japan).

#### 2.6.6 X-ray diffraction

X-ray diffraction (XRD) analysis of UF3 before and after glycosylation was performed following the method described by (Zhu et al., 2024). The measurement was conducted using an X-ray diffractometer (X'Pert PRO, PANalytical, the Netherlands) with a scanning range of 5° to 80° (2θ) at a scanning rate of 2°/min.

### 2.7 Determination of particle size and zeta potential

Following the method of Geng et al. (2021) with minor modifications, the sample was prepared into a 5 mg/mL test solution using ultrapure water. A 1 mL aliquot of the test solution was taken for measurement of the average particle size and zeta potential of each sample using a nanoparticle size and zeta potential analyzer (ZETASIZER NANO ZSE, Malvern Panalytical, UK) at room temperature. Each sample was measured in triplicate.

### 2.8 Differential scanning calorimetry

Approximately 5 mg of the sample was weighed and placed in an aluminum pan, with an empty aluminum pan used as a reference. The thermal analysis was performed within a scanning temperature range of 20 °C to 200 °C at a heating rate of 10 °C/min. The air flow rate was maintained at 20 mL/min throughout the measurement.

### 2.9 Determination of surface hydrophobicity and solubility

Surface hydrophobicity was determined using the bromophenol blue (BPB) binding method (Zeng et al., 2024). Briefly, 1 mL of sample solution (5 mg/mL) was mixed with 200 μL of BPB (1 mg/mL in PBS), vortexed, allowed to stand for 10 min, and centrifuged at 5000 rpm for 15 min. The supernatant was diluted 10-fold, and absorbance was measured at 595 nm. BPB bound was calculated as (2–4):

(2-4) $$\mathrm{BPB}\;\text{Bound}\;\left(\mathrm{\mu g}\right)=200\times \frac{A_{\mathrm{blank}}-A_{\mathrm{sample}}}{A_{\mathrm{blank}}}$$

In the formula: *A*_blank_ is the absorbance of the PBS control at 595 nm; *A*_sample_ is the absorbance of UF3 or GUF3 at 595 nm.

Solubility was assessed following Zhao et al. (2024). Samples (1 mg/mL) were centrifuged at 10,000 rpm for 10 min. Protein content in the supernatant was determined using a BCA kit (Beyotime, Shanghai, China). Solubility was calculated as **(2–5)**:

(2-5) $$\text{Solubility}\;\left(\%\right)=\frac{C}{C_{0}}\times 100\%$$

In the formula: *C* is the protein content in the supernatant; *C*_0_ is the total protein content in the sample.

### 2.10 Hypolipidemic activity of UF3 and GUF3 in HepG2 cells

#### 2.10.1 HepG2 cells culture

HepG2 cells (Cell Bank of the Chinese Academy of Sciences, Shanghai, China) were cultured in DMEM medium supplemented with 10% fetal bovine serum and 1% penicillin-streptomycin, and maintained in a 37 °C incubator with 5% CO₂. When the cell confluence reached approximately 80%, the culture medium was removed, and the cells were gently rinsed with PBS, digested with 0.25% trypsin (containing EDTA), and subcultured at a 1:2 ratio. All experiments were conducted using cells in the logarithmic growth phase with good viability.

#### 2.10.2 Cytotoxicity of UF3 and GUF3 in HepG2 cells

HepG2 cells with good growth status were seeded into a 96-well plate at a density of 1 × 10^4^ cells per well. After 24 h of culture, the original medium was removed, and the cells were treated with UF3 or GUF3 at concentrations ranging from 0 to 2000 μg/mL (specifically 0, 2000, 1000, 400, 200, 100, 50, and 25 μg/mL) for an additional 24 h. Cell viability then was determined using a CCK-8 kit (Abbkine Scientific Co., Ltd., Wuhan, China). Briefly, the culture medium was discarded, and 100 μL of serum-free medium containing 10% CCK-8 reagent was added to each well in the dark. After 1 h of incubation, the absorbance at 450 nm was measured using a microplate reader. Cell viability was calculated according to Formula (2-6), with *n* = 4 replicates per group.

(2-6) $$\text{Cell viability}\;\left(\%\right)=\frac{\mathrm{Experimental well}\;\mathrm{OD}\;\mathrm{value}-\mathrm{blank well}\;\mathrm{OD}\;\mathrm{value}}{\mathrm{Control well}\;\mathrm{OD}\;\mathrm{value}-\mathrm{blank well}\;\mathrm{OD}\;\mathrm{value}}$$

#### 2.10.3 Oil red O staining

Based on preliminary experiments, 700 μmol/L free fatty acids (FFA, oleic acid: palmitic acid = 2:1, molar ratio) was selected as the modeling inducer, and 10 μmol/L simvastatin (SIM) was used as the positive control. Following the method described by Dong et al. (2024) and the instructions of the Oil Red O staining kit (Solarbio Science & Technology Co., Ltd., Beijing, China), HepG2 cells were first treated with the FFA and respective samples for 24 h, and then subjected to Oil Red O staining. After microscopic observation, 0.6 mL of 60% isopropanol was added to dissolve the stained lipid droplets for 20 min, and the absorbance at 510 nm was measured.

#### 2.10.4 Determination of TC and TG contents

The contents of total cholesterol (TC) and triglycerides (TG) in HepG2 cells treated with FFA and the respective samples for 24 h were measured according to the instructions of the TC and TG assay kits (Beijing Pulilai Gene Technology Co., Ltd., Beijing, China).

### 2.11 In vitro simulated digestive characteristics

Simulated gastrointestinal digestion was performed following Sheng et al. (2025) with modifications. Samples (100 mg of UF3 or GUF3) were dissolved in 10 mL of artificial gastric juice (pH 2.0 ± 0.5, containing pepsin) and incubated at 37 °C in a water bath shaker (120 rpm). Aliquots were collected at 1 and 2 h, heat-inactivated (90 °C, 10 min), and centrifuged. The supernatant was adjusted to neutral pH and then and used for enzyme inhibitory activity assays.

For intestinal digestion, the remaining gastric digest was mixed with an equal volume of artificial intestinal juice (containing pancreatin in phosphate buffer) and incubated at 37 °C (120 rpm). Aliquots were taken at 1, 2, 3, and 4 h, heat-inactivated, centrifuged, and the supernatant was collected for activity determination.

### 2.12 Statistical analysis

Statistical analysis was performed using SPSS 27.0 software. A homogeneity of variances test was first conducted. For comparisons between two groups, an independent samples *t*-test was used, while one-way ANOVA followed by Duncan's post hoc analysis was applied for multiple group comparisons, with statistical significance set at *p* < 0.05. All experiments were performed in triplicate, and data are presented as mean ± standard deviation. Figures were generated using GraphPad Prism 9.

## 3 Results and discussion

### 3.1 Inhibitory effects of oyster hydrolysate and its ultrafiltration fractions on PL and CE

**Fig. 1:**
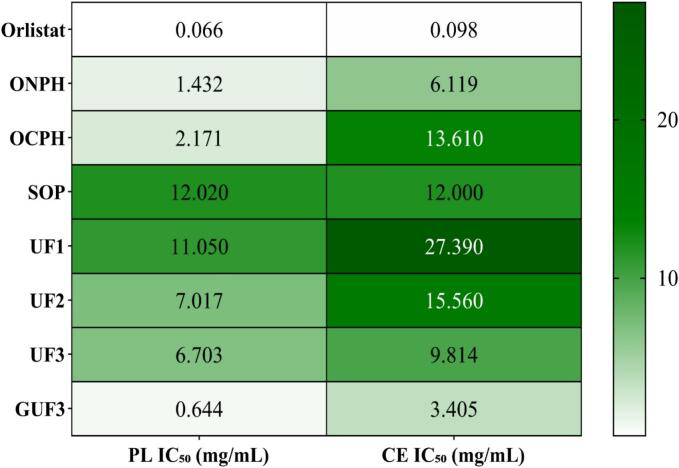
IC₅₀ values of oyster hydrolysate, ultrafiltration fractions, and glycosylation products against pancreatic lipase and cholesterol esterase.

PL and CE are two important lipid digestive enzymes. PL is responsible for the hydrolysis of dietary triglycerides into easily absorbed free fatty acids and monoacylglycerols, while CE hydrolyzes cholesterol esters into free cholesterol (Shen et al., 2023). The excessive absorption of these lipids can lead to metabolic disorders and increase the risk of cardiovascular diseases (Shen et al., 2023). Studies have suggested that the overactivation of PL and CE is a significant factor contributing to hyperlipidemia and obesity. Therefore, targeted inhibition of these enzymes' activity to reduce the intestinal absorption of small molecule lipids like free fatty acids and free cholesterol is a potential strategy for effectively alleviating hyperlipidemia and obesity (Mudgil et al., 2022). The inhibitory activities of oyster neutral protease hydrolysate (ONPH), oyster compound protease hydrolysate (OCPH), and SOP against PL and CE are shown in **Fig. S1 (A–D) and** Fig. 1**.** ONPH, OCPH, and SOP all exhibited dose-dependent inhibitory activity against both PL and CE within the concentration range of 0–20 mg/mL. Among them, ONPH demonstrated the best inhibitory activity against PL and CE, with IC₅₀ values of 1.43 mg/mL and 6.119 mg/mL, respectively. This was followed by OCPH, with IC₅₀ values of 2.171 mg/mL and 13.61 mg/mL, respectively, while SOP showed the weakest inhibition. The variation in their inhibitory effects on PL and CE is likely attributable to the different types of peptides generated by hydrolysis with different proteases, which possess distinct PL and CE inhibitory properties (Mudgil et al., 2018). However, compared to the positive control Orlistat, the inhibitory efficacy of ONPH still showed some gap. Orlistat had IC₅₀ values of 0.067572 mg/mL for PL and 0.09769 mg/mL for CE. This might be because ONPH is inferior to orlistat in terms of the binding precision to the enzyme's active site, interaction strength, and component purity. Nevertheless, as a naturally derived functional factor, ONPH offers higher safety and fewer side effects. It may have greater application potential than chemically synthesized drugs in the development of functional foods or products for auxiliary lipid-lowering and anti-obesity purposes. As reported by Mudgil et al. (2022)., the hydrolysates from cow and camel casein enzymolyzed for 3 h had IC₅₀ values for PL of 1.88 mg/mL and 1.25 mg/mL, respectively. This further indicates that natural food-derived peptides, as mild and safe functional factors, hold significant potential for development in alleviating hyperlipidemia, and managing obesity and related metabolic diseases.

The above results showed that ONPH exhibited higher inhibitory activity against both PL and CE compared to OCPH and SOP. However, given the complex composition of its enzymatic hydrolysate, ultrafiltration was employed to preliminarily isolate and purify ONPH, aiming to screen for components with greater hypolipidemic potential. The inhibitory effects of the various ultrafiltration fractions on PL and CE were then evaluated. As shown in **Fig. S1 (E–K) and** Fig. 1, the UF3 fraction (<3 kDa) demonstrated superior inhibitory activity against both PL and CE compared to the UF2 (3–5 kDa) and UF1 (5–8 kDa) fractions. The IC₅₀ values of UF3 for PL and CE were 6.703 mg/mL and 9.814 mg/mL, respectively. This could be attributed to the more flexible spatial structure of smaller molecular weight peptides, which may facilitate easier binding to the active centers of PL and CE (Huang et al., 2025b; Tang & Koh, 2023). Notably, although UF3 showed superior inhibition compared to the other fractions, its IC₅₀ values were still higher than those of the unpurified ONPH. This may be due to the enrichment of small molecular peptides by ultrafiltration, while also removing some of the active substances that have synergistic effects with these peptides, thereby leading to a slight decrease in the overall inhibitory activity. However, ONPH has a complex composition, whereas UF3 offers advantages such as more uniform molecular weight, higher purity, better solubility, and sufficient exposure of reactive groups, making it an ideal substrate for structural modification. Therefore, UF3 was selected for subsequent glycosylation modification to further improve its biological activity and physicochemical properties.

### 3.2 Effect of glycosylation products on PL and CE

Under the catalysis of TGase, the free amino group (acyl receptor) of GlcN can conjugate with acyl donors (such as Gln) on the polypeptide chain to form glycopeptides (GPs) (Liu et al., 2022). Previous studies have shown that GPs exhibit superior physicochemical characteristics and bioactivities than conventional peptides (Moradi et al., 2016). This study investigated the effects of different reaction conditions on the DG of the glycosylation product (GUF3) and its inhibitory activities against PL and CE. **Fig. S2 (A)** shows that in the temperature range of 30 to 50 °C, the DG of GUF3 gradually increases with the rise in reaction temperature and eventually tends to be gentle, reaching a maximum of 27.2%. This may be due to the loosening of the peptide chain structure caused by temperature changes, which exposes reactive sites on the peptide chain and increases the collision frequency between the enzyme and the substrate. However, when the temperature becomes too high, DG no longer increases significantly, which may be attributed to the reduced enzyme activity of TGase at elevated temperatures (Liu et al., 2025). Notably, the inhibitory activity of GUF3 on PL and CE did not increase with the rise of DG after reaching the peak at 40 °C. At this time, PLI and CEI were 86.7% and 85.9%, respectively, which were significantly higher than those of UF3 and GlcN mixture before reaction (PLI and CEI: 59.7% and 62.3%, respectively) (*p* < 0.05). This result indicates that moderate glycosylation can significantly enhance the inhibitory activities of GUF3 against PL and CE. This may be attributed to the introduction of GlcN, which alters the spatial conformation and charge distribution of the peptide chain (Zhu et al., 2024), making it more accessible to the active centers of PL and CE. Additionally, the sugar chain structure of GlcN itself may synergistically interfere with the enzyme's active sites along with the polypeptide, thereby enhancing the inhibitory effect. However, when DG is too high due to high temperature, the molecular structure of the product may become too complex or large due to excessive glycosylation, which hinders the effective contact between GUF3 and the PL and CE active sites, resulting in no increase or even decrease in inhibitory activity (Wang et al., 2016). Similarly, the DG of GUF3 and its inhibitory activity against PL and CE showed an initial increase followed by stabilization or decline with prolonged reaction time, decreased sugar-to-peptide ratio, and increased TGase addition. All parameters peaked at 4 h reaction time, 2:1 sugar-to-peptide ratio, and 15 U/g TGase addition. Both the DG of GUF3 and its inhibitory activity against PL and CE exhibited a threshold, meaning they did not continue to increase with prolonged reaction time or higher amounts of sugar or TGase added. This may be after the reaction reaches a certain stage, the depletion of the substrate and the reduction of the binding sites on the polypeptide chain make the system reach a dynamic equilibrium (Liu et al., 2025). Under equilibrium conditions, further increasing reaction time, sugar, or TGase content fails to enhance the effective glycosylation reaction. Moreover, excessive sugar or TGase may lead to the formation of sugar condensates or over-crosslinked products, causing excessive steric hindrance, which not only hinders glycosylation but also alters the product's structure, consequently affecting its inhibitory activity against PL and CE (Zhao et al., 2026). Therefore, to obtain GUF3 with optimal PL and CE inhibitory activity, this study established the optimal reaction conditions as: temperature 40 °C, reaction time 4 h, a molar ratio of GlcN to peptide of 2:1, and TGase addition 15 U/g. Under these optimized conditions, the prepared GUF3 exhibited IC₅₀ values against PL and CE of 0.644 mg/mL and 3.405 mg/mL, respectively **(**Fig. 1**)**, which were lower than those of UF3. This result indicates that the optimized glycosylation modification can significantly enhance the enzyme inhibitory activity of oyster peptides.

**Fig. 2:**
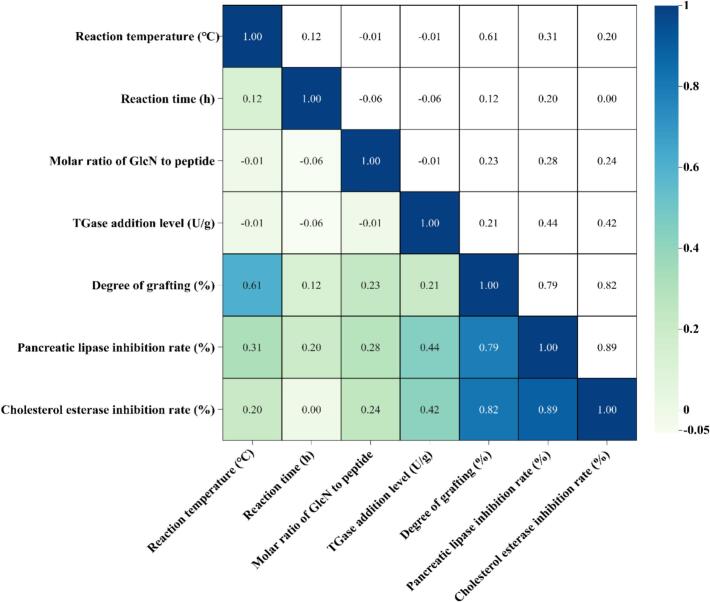
Correlation analysis between different reaction conditions and the degree of grafting, as well as the inhibitory activities against pancreatic lipase and cholesterol esterase of glycosylation products.

Spearman rank correlation analysis further revealed **(**Fig. 2**)** that DG was significantly and strongly positively correlated with both PLI and CEI (*r* = 0.79–0.82), indicating that the DG of glycosylation is a key factor in enhancing the inhibitory activity of UF3 against PL and CE. However, among the single factors, the reaction temperature exhibited a relatively stronger influence on DG, while the effects of reaction time, molar ratio of GlcN to peptide, and TGase addition level were comparatively weaker. This may be attributed to the fact that temperature significantly affects both TGase activity and substrate conformation, whereas the other factors, upon reaching their reaction saturation points, tend to plateau (Liu et al., 2025). Continued variation in these single factors fails to substantially alter the reaction process, thus showing weaker influences in the correlation analysis.

### 3.3 Structural characterization of glycosylation products

#### 3.3.1 Molecular weight analysis

**Fig. 3:**
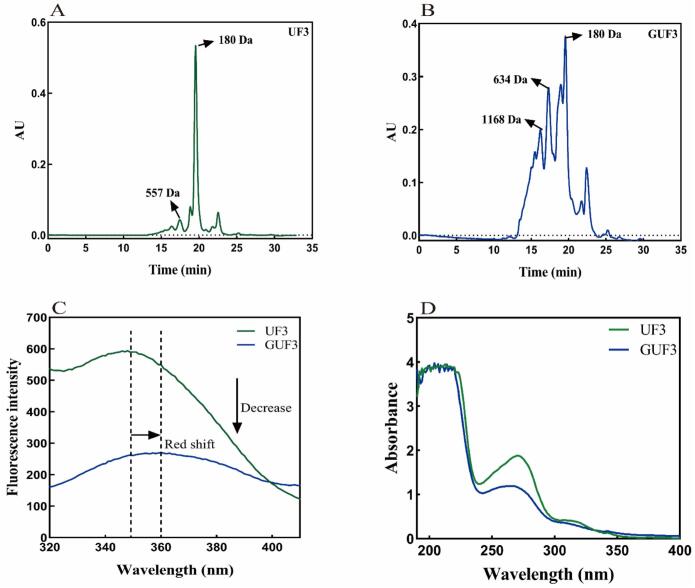
Structural characteristics of the UF3 before and after glycosylation (A: Molecular weight distribution of UF3; B: Molecular weight distribution of GUF3; C: Intrinsic fluorescence spectroscopy; D: UV–Vis absorption spectrum).

In the presence of GlcN, UF3 underwent glycosylation catalyzed by TGase to form GUF3. High-performance gel chromatography was employed to analyze the molecular weights of the products before and after glycosylation. The separation principle of this method is that substances with larger molecular weight are not easy to enter the microporous structure inside the gel particles due to the volume exclusion effect, thus are eluted earlier (Fukudome et al., 2021). As shown in the chromatogram of Fig. 3A–B, compared with UF3, the peak area of GUF3 in the shorter retention time region significantly increased. This indicates that under the catalysis of TGase, GlcN is covalently coupled with the peptide chains of UF3, forming glycosylated products with higher molecular weight. Similar results have been reported by Chen et al. (2019) in the glycosylation modification of whey protein isolates.

#### 3.3.2 Intrinsic fluorescence spectroscopy analysis

The peptide chain contains various fluorescent substances, such as tyrosine (Tyr), tryptophan (Trp), and phenylalanine (Phe). Their fluorescence properties are highly sensitive to microenvironmental changes and can serve as probes for spatial and structural alterations in the peptide chain (Liu et al., 2025). As shown in Fig. 3C, the maximum emission wavelength (λ_max_) of GUF3 exhibited a red shift (from 349 to 360 nm). This indicates that glycosylation induces structural stretching of the peptide chain, exposing Trp residues—which might otherwise be buried within hydrophobic regions of the chain—to a more polar solvent environment (Zhao et al., 2024). In addition, compared with UF3, the fluorescence intensity of GUF3 significantly decreased. This may be due to the covalent conjugation of GlcN to the peptide chain of UF3, which altered the microenvironment around the Trp residues, thereby leading to fluorescence quenching. It could also be attributed to the steric hindrance effect introduced by the sugar chain, which may shield the fluorescent chromophores and further reduce the fluorescence intensity (Zhao et al., 2026). The results indicate that GlcN was successfully conjugated to UF3 catalyzed by TGase, leading to changes in the spatial conformation of UF3. This is consistent with the report by Parandi et al. (2024). They found that after the glycosylation reaction between sesame protein hydrolysate and gum arabic, the maximum peak of its fluorescence spectrum showed a red shift accompanied by fluorescence quenching, indicating that during glycosylation, the Trp residues migrated from a hydrophobic environment to a more hydrophilic microenvironment.

#### 3.3.3 UV–Vis spectroscopic analysis

Due to the presence of numerous chromophores within the polypeptide chain, peptide molecules exhibit a typical UV absorption peak around 280 nm. Structural changes in the peptide molecule may lead to variations in the UV absorbance value to different extents (Liu et al., 2025). As shown in Fig. 3D, UF3 exhibited distinct absorption peaks near 280 nm. In contrast, the glycosylated product (GUF3) showed a significant decrease in UV absorption intensity. This is likely because the covalent conjugation between the sugar chain and the peptide chain introduced steric hindrance, which masked some chromophores of the Tyr and Trp residues, thereby leading to a decrease in absorbance (Zhu et al., 2024).

#### 3.3.4 FT-IR spectroscopy analysis

**Fig. 4:**
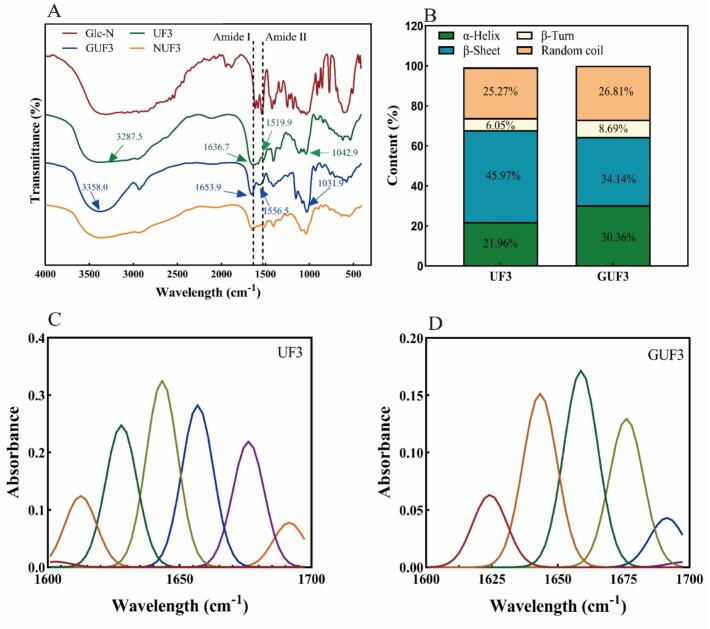
Analysis of the FT-IR of UF3 before and after glycosylation (B-D: 1700–1600 cm^−1^; E: secondary structure percentage (%)); NUF3 is a mixture of UF3 and GlcN.

The glycosylation reaction between Gln and peptide molecules catalyzed by TGase may lead to structural changes in the polypeptide. FT-IR spectroscopy can analyze structural changes in polypeptides based on shifts in the position, intensity, and shape of characteristic absorption peaks in the infrared region (Chang et al., 2022). Therefore, this study utilized FT-IR to evaluate the structural changes of UF3 before and after glycosylation **(**Fig. 4A–D**)**. In the FT-IR spectrum, the amide I band (1700–1600 cm^−1^) primarily caused by the C=O stretching vibration in the peptide backbone, reflects the secondary structure type of the peptide chain through its peak position and intensity (β-sheet corresponds to 1640–1610 cm^−1^ and 1680–1670 cm^−1^, α-helix corresponds to 1660–1650 cm^−1^, random coil corresponds to 1650–1640 cm^−1^, β-turn corresponds to 1670–1660 cm^−1^ and 1680–1700 cm^−1^) (Long et al., 2015). The amide II band (1600–1500 cm^−1^) is associated with N—H bending vibration and C—N stretching vibration.

The FT-IR results indicate that compared to the original UF3, the amide I band absorption peak of GUF3 shifted blue from 1636.7 cm^−1^ to 1653.9 cm^−1^, and the peak shape became sharper and more symmetrical. This is likely due to the -OH and -NH₂ groups introduced by GlcN forming strong intramolecular hydrogen bonds with the peptide chain, altering the electron cloud density around C=O and leading to a blue shift (Yang et al., 2023a). The amide II band absorption peak of GUF3 shifted blue from 1519.9 cm^−1^ to 1556.5 cm^−1^, further indicating that glycosylation altered the secondary structure of UF3. Moreover, the absorption peak of GUF3 near 3500 cm^−1^ became stronger, likely due to the introduction of -OH from GlcN, which enhanced hydrogen bonding effects. This is consistent with the findings reported by Zhao et al. (2024). The absorption peak in the 1200–950 cm^−1^ wavenumber range represents the characteristic absorption peak of covalent binding between glycosyl groups and polypeptides (Zhang et al., 2024). The absorption peak of GUF3 in this range became broader and stronger, demonstrating that GlcN was successfully covalently conjugated to the peptide chain, resulting in an increased number of C—N covalent bonds (Zheng et al., 2022).

Fourier deconvolution, peak fitting and integral processing were performed on the amide I band. The results **(**Fig. 4B**)** showed that the relative content of β-sheet in UF3 was 45.97%, and that of α-helix was 21.96%. In contrast, for GUF3, the α-helix content increased to 30.36%, while the β-sheet content decreased to 34.14%. The β-turn content slightly increased (from 6.05% to 8.69%), with minimal change in random coil. This result is similar to that reported by Shang et al. (2020) for xylose-glycosylated whey protein isolate (WPI), where glycosylation modification of WPI resulted in a decrease in β-sheet content and an increase in α-helix content. Furthermore, this quantitative result aligns with the trend of peak shifts observed in the amide I band, indicating that TGase-catalyzed GlcN glycosylation altered the secondary structure of UF3, promoting a transition from β-sheet to α-helix. This change may make it less susceptible to denaturation caused by external environmental factors such as temperature and pH variations (Jiang et al., 2024; Song et al., 2016).

#### 3.3.5 Microstructure analysis

**Fig. 5:**
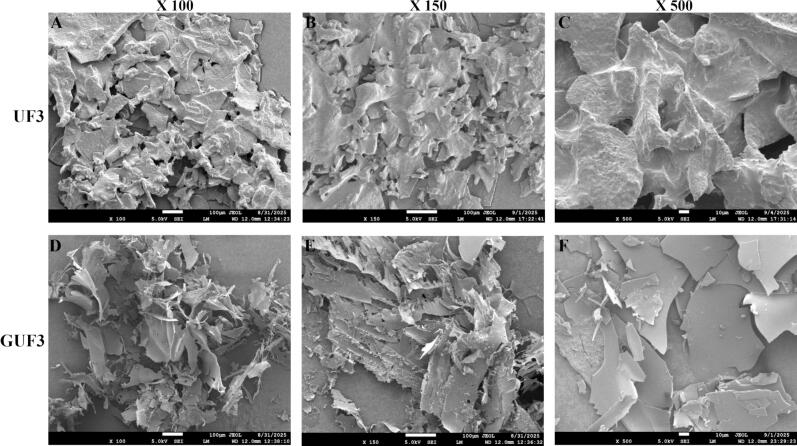
Microstructure observation of UF3 before and after glycosylation (A-C: SEM images of UF3; D-F: SEM images of GUF3, at magnifications of 100×, 150×, and 500×).

SEM was employed to characterize the microstructures of UF3 before and after glycosylation. As shown in Fig. 5, before glycosylation, UF3 exhibited a rough, thick, and irregularly stacked lamellar structure. In contrast, after TGase-catalyzed GlcN glycosylation, the microstructure changed significantly, with the surface of GUF3 presenting a looser, smoother, and thinner filmy network and scaly morphology. This may be attributed to the introduction of hydrophilic sugar chains, which reduced the rigid aggregation and tight stacking between peptide chains (Li et al., 2024; Zhu et al., 2024). This structural change may enhance the interaction of GUF3 with water, thereby improving its solubility and dispersibility in aqueous solutions (Li et al., 2024).

#### 3.3.6 X-ray diffraction analysis

**Fig. 6:**
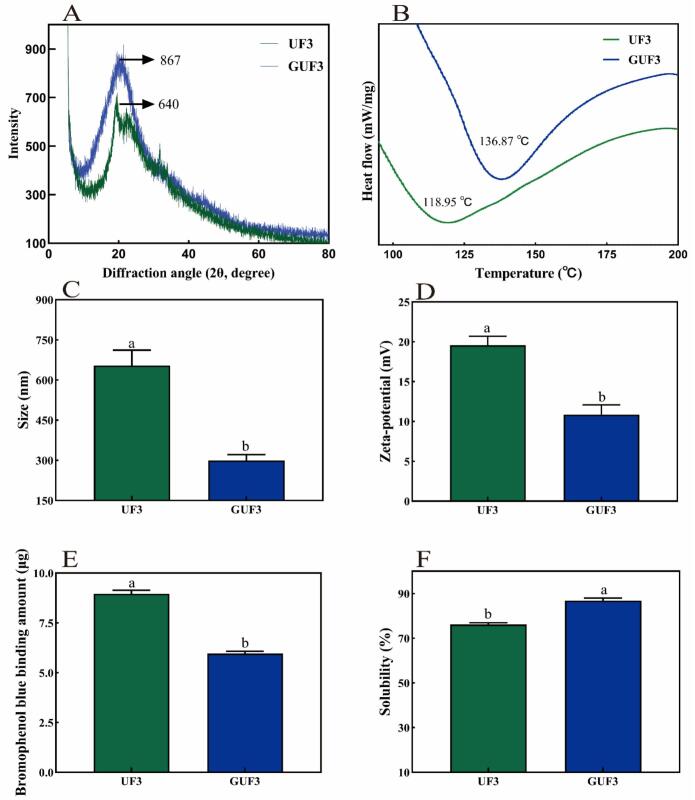
(A) XRD patterns; (B) DSC curves; (C) Average particle size; (D) Zeta potential values; (E) Surface hydrophobicity; (F) Solubility. Data are presented as mean ± standard deviation (*n* = 3). Different lowercase letters indicate statistically significant differences (*p* < 0.05).

XRD was used to analyze the crystalline structures of UF3 and GUF3, with a scanning range of 5°–80° (2θ). As shown in Fig. 6A, both UF3 and GUF3 exhibited characteristic diffraction peaks near 2θ ≈ 20°, indicating the presence of crystalline structures. The diffraction peak intensity of UF3 was 640. After glycosylation modification, the peak intensity increased to 867, and the peak shape became sharper and more symmetrical. These changes suggest that glycosylation enhanced the crystallinity of UF3 and likely improved its local structural order (Zhu et al., 2024).

### 3.4 Particle size and zeta potential analysis

The average particle size is one of the key indicators reflecting the structural changes and aggregation behavior of peptide chains, and its size significantly influences the functional properties of peptides. As shown in Fig. 6C, the average particle sizes of UF3 and the glycosylated product GUF3 were 654.2 ± 57.6 nm and 300.0 ± 21.5 nm, respectively. The results indicated that after glycosylation modification, the average particle size of GUF3 decreased significantly by 54.2% compared to the original UF3 (*p* < 0.05). This phenomenon suggests that the glycosylation of the peptide chains with GlcN, catalyzed by TGase, may have increased their steric hindrance or hydrophilicity, thereby reducing their aggregation behavior (Li et al., 2024). This observation is consistent with the SEM results that GUF3 exhibited a looser and thinner sheet-like morphology.

However, compared to UF3, the absolute values of the Zeta potential for GUF3 were significantly reduced (*p* < 0.05) (see Fig. 6D). This may be attributed to GlcN, as a cationic monosaccharide, whose introduction partially neutralizes the negative charges on the peptide chain surface. Meanwhile, glycosylation modification alters the surface charge distribution and induces a shielding effect. This phenomenon is similar to the findings reported by Zhu et al. (2024) regarding TGase-catalyzed GlcN-modified ovalbumin.

### 3.5 Thermal stability analysis

DSC was used to assess the thermal stability of UF3 and GUF3 within the temperature range of 20–200 °C. Generally, a higher denaturation temperature (Td) value indicates better thermal stability of the sample (Meng et al., 2023). As shown in Fig. 6B, the Td value of UF3 was 118.95 °C, while that of GUF3 increased to 136.87 °C, indicating that GUF3 exhibited enhanced thermal stability. This improvement may be attributed to the covalent conjugation of GlcN with UF3, where the steric hindrance of the sugar chains inhibits heat-induced aggregation and structural disruption of the polypeptide chains (Li et al., 2024). These results are consistent with the findings reported by Jiang et al. (2024), in which TGase-catalyzed carboxymethyl chitosan modification enhanced lactoferrin's thermal stability. The results show that TGase-catalyzed glycosylation reactions can effectively improve the thermal stability of polypeptide chains.

### 3.6 Surface hydrophobicity and solubility analysis

The amount of BPB bound can reflect the surface hydrophobicity (H_0_) of UF3 before and after glycosylation. As shown in Fig. 6E, compared to the H_0_ value of UF3 (8.96 ± 0.18 μg), that of the glycosylated product GUF3 decreased significantly to 5.96 ± 0.12 μg (*p* < 0.05). This indicates that the introduction of GlcN effectively reduces the H_0_ of the peptide chain. This may be attributed to the covalent conjugation of GlcN to UF3 catalyzed by TGase, which not only introduces abundant hydrophilic hydroxyl and amino groups, significantly reducing the proportion of hydrophobic groups on the peptide surface, but also forms a more stable hydrophilic protective layer on the surface (Jiang et al., 2024; Yang et al., 2019). Consequently, this modification effectively inhibits the aggregation behavior caused by hydrophobic interactions between peptide chains (Li et al., 2024).

Solubility is one of the most fundamental functional properties of proteins and significantly influences other physicochemical characteristics, functional attributes, and bioactivity. Under pH = 7 conditions, the solubility values of UF3 and GUF3 were 76.1% and 86.9%, respectively **(**Fig. 6F**)** (*p* < 0.05). GUF3 exhibits higher solubility, which may be attributed to the covalent conjugation of GlcN to the peptide surface, introducing increased steric hindrance that reduces aggregation between particles (Qu et al., 2018). Moreover, the increased number of hydrophilic groups and enhanced hydrogen-bonding capacity of the conjugate further improve the interaction between the peptide and water (Yang et al., 2023b). These observations are consistent with the previous SEM results showing a loose network morphology and lower aggregation degree of GUF3, as well as the significantly reduced average particle size and surface hydrophobicity. In conclusion, the TGase-catalyzed glycosylation of UF3 with GlcN significantly enhances its solubility.

### 3.7 Hypolipidemic effects of glycosylated products on FFA-induced HepG2 cells

**Fig. 7:**
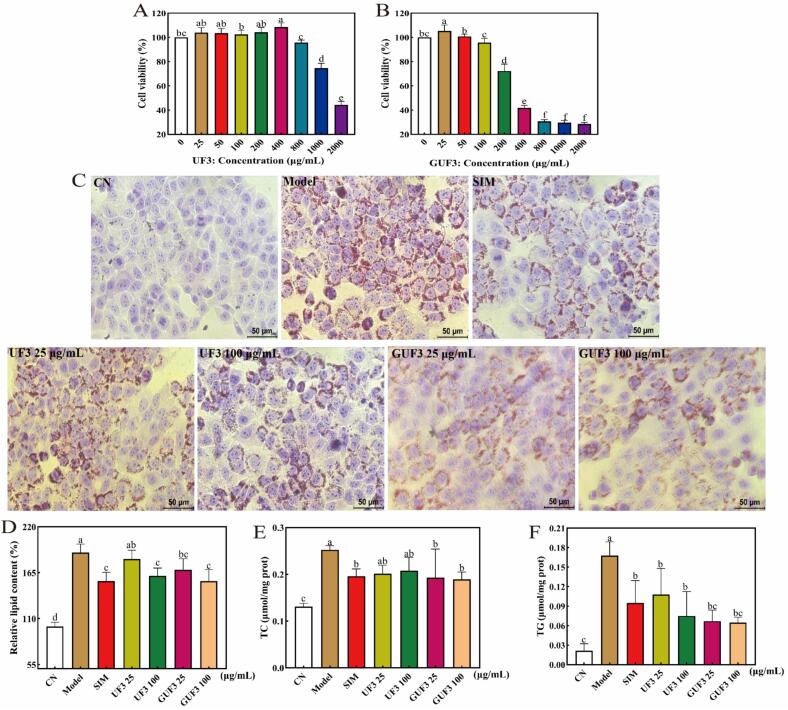
Concentration screening and hypolipidemic effects of UF3 and GUF3. (A: Cell viability of UF3; B: Cell viability of GUF3; C: Microscopic observation of Oil Red O staining; D: Quantitative analysis of Oil Red O staining; E: TC content; F: TG content). Data are presented as mean ± standard deviation (n = 3). Different lowercase letters indicate statistically significant differences (*p* < 0.05). Note: Oil Red O staining images were acquired at ×40 original magnification (scale bar = 50 μm). (For interpretation of the references to colour in this figure legend, the reader is referred to the web version of this article.)

The CCK-8 assay results **(**Fig. 7A–B**)** showed that UF3 and GUF3 exhibited cell viability exceeding 95% at concentrations up to 100 μg/mL, indicating no cytotoxic effects on HepG2 cells. Therefore, 25 and 100 μg/mL were selected as the low- and high-dose concentrations for subsequent experiments. The experimental design included a control group (without FFA or samples, CN), a model control group (FFA), experimental groups (FFA with 25 or 100 μg/mL UF3 or GUF3), and a positive control group (FFA with 10 μmol/L SIM).

The results of Oil Red O staining **(**Fig. 7C**)** showed that compared with the CN group, a large number of red granular lipid droplets accumulated in HepG2 cells of the Model group, while interventions with various concentrations of UF3 and GUF3 significantly reduced intracellular lipid droplet accumulation. Notably, GUF3 exhibited a superior lipid-lowering effect compared to UF3 at both tested concentrations **(**Fig. 7D**)**. Specifically, compared with the Model group, the absorbance values in the 25 and 100 μg/mL GUF3 groups decreased by 10.9% (*p* < 0.05) and 18.2% (*p* < 0.05), respectively, outperforming the 4.1% and 14.7% reductions (*p* < 0.05) observed with UF3, with a particularly pronounced advantage at 25 μg/mL, although the difference between the two treatment groups at 100 μg/mL did not reach statistical significance.

The TC and TG assay results further indicated **(**Fig. 7E–F**)** that compared with the CN group, intracellular TC and TG levels were significantly elevated in the Model group (*p* < 0.05), which further indicated that FFA successfully induced lipid metabolism disorder in HepG2 cells. Both UF3 and GUF3 interventions reduced intracellular TC and TG content to varying degrees. Among them, compared with the Model group, the TC levels in the 25 and 100 μg/mL GUF3 groups decreased by 23.7% (*p* < 0.05) and 25.1% (*p* < 0.05), respectively, outperforming the 20.2% (*p* > 0.05) and 17.7% (*p* > 0.05) reductions observed in the UF3 groups. Similarly, GUF3 intervention reduced TG levels by 60.0% (*p* < 0.05) and 61.6% (*p* < 0.05) at the same concentrations, compared to 36.0% (*p* < 0.05) and 55.2% (*p* < 0.05) for UF3, both of which were lower than those achieved by GUF3.

These results demonstrate that both UF3 and GUF3 can inhibit FFA-induced lipid accumulation in HepG2 cells, with GUF3 exhibiting stronger hypolipidemic effects than UF3. This finding is consistent with the results in Section 3.2, where GUF3 showed superior inhibitory activity against PL and CE compared to UF3, further indicating that TGase-mediated GlcN glycosylation helps enhance the hypolipidemic activity of peptides. This may be attributed to the introduction of sugar groups, which improved the physicochemical properties (such as solubility) of the peptides and enhanced their ability to penetrate cell membranes (Moradi et al., 2016), thereby more effectively regulating intracellular lipid metabolism and strengthening their capacity to clear lipid droplets and suppress lipid accumulation. Additionally, it might also be due to the synergistic effect between the inherent lipid-lowering activity of GlcN and that of peptides. Studies have shown that GlcN can modulate hepatic lipid synthesis and alleviate the progression of metabolic dysfunction-associated fatty liver disease (Ryu et al., 2025). Under high-glucose or energy-surplus conditions, GlcN can also regulate the expression of lipid synthesis-related genes in HepG2 cells and significantly inhibit lipid accumulation (Park et al., 2020). Therefore, the covalent conjugation of GlcN with UF3 further enhances the hypolipidemic efficacy.

### 3.8 In vitro simulated digestion characteristics

**Fig. 8:**
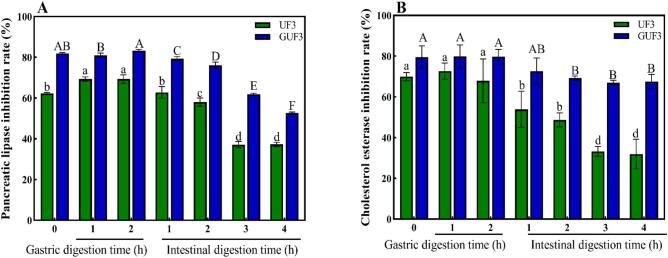
Effect of in vitro simulated digestion products on the inhibition rate of pancreatic lipase and cholesterol esterase (A: Inhibition rate of pancreatic lipase; B: Inhibition rate of cholesterol esterase). Data are presented as mean ± standard deviation (n = 3). Different lowercase or uppercase letters indicate statistically significant differences (*p* < 0.05).

After gastrointestinal digestion, bioactive peptides may undergo structural alterations, which can subsequently affect their biological activity (Mirzaei et al., 2020). As shown in Fig. 8, the inhibitory activity of UF3 and GUF3 against PL and CE after gastrointestinal digestion showed that both retained relatively high inhibitory activity during the gastric digestion phase. This is likely due to their strong resistance to the acidic gastric environment and the hydrolytic action of pepsin, which preserved their active sequences. However, upon entering the intestinal digestion phase, the inhibitory activities of UF3 and GUF3 against PL and CE significantly decreased (*p* < 0.05), dropping to 37.3% and 52.6% (for PL inhibition), and 32.0% and 67.5% (for CE inhibition) after 4 h, respectively. This decline may be attributed to the stronger hydrolytic specificity of trypsin in the digestive fluid, which partially degraded the active peptide fragments (Qian et al., 2022). Notably, the decrease in activity for GUF3 was smaller than that for UF3. This difference may be due to the steric hindrance effect introduced by GlcN, as well as the more rigid structure formed between the sugar moiety and the peptide chain, both of which increase the difficulty of enzymatic hydrolysis by digestive enzymes (Huang et al., 2025b; Wu et al., 2022). Additionally, the introduction of GlcN may alter the physicochemical properties of the peptide chain, thereby affecting its interaction with digestive enzymes (Huang et al., 2025b). Wang et al. (2020) also reported a similar phenomenon, showing that glycosylated corn peptides exhibited stronger resistance to gastrointestinal digestive enzymes and could protect alcohol dehydrogenase from oxidative inactivation. Collectively, these results indicate that TGase-catalyzed GlcN glycosylation modification can enhance the digestive stability of active peptides and reduce activity loss.

## 4 Conclusion

This study successfully prepared the glycosylated oyster peptide GUF3 via transglutaminase (TGase)-catalyzed glucosamine (GlcN) modification of the oyster hydrolysate ultrafiltration fraction UF3. Glycosylation induced a secondary structural shift of UF3 from β-sheet to α-helix, reduced molecular aggregation, and remodeled its microstructure into a smooth, hydrophilic thin-film network. These structural changes significantly improved UF3's physicochemical properties, including elevated solubility and thermal stability, reduced particle size and surface hydrophobicity. More importantly, this structural enhancement enhanced GUF3's resistance to enzymatic hydrolysis during gastrointestinal digestion, effectively preserving its bioactivity in the intestinal phase. Consequently, GUF3 exhibited remarkably enhanced in vitro hypolipidemic activity, with higher pancreatic lipase and cholesterol esterase inhibition, and superior efficacy in alleviating free fatty acid-induced lipid accumulation in HepG2 cells compared with unmodified UF3. Collectively, TGase-catalyzed GlcN glycosylation is a promising mild modification strategy that synchronously improves the physicochemical properties, digestive stability and hypolipidemic activity of oyster peptides, providing a solid theoretical basis for its development as a natural hypolipidemic ingredient in marine functional foods.

Limitations exist in the current in vitro and simulated digestion studies. HepG2 cells provide an ideal model for preliminary screening and mechanistic investigation, yet they cannot fully recapitulate the complex physiological environment in vivo. Thus, further in vivo animal studies are still needed to validate the actual hypolipidemic efficacy and metabolic regulatory mechanism of GUF3. Relevant in vivo studies are currently underway in our laboratory and will be reported separately in future work. Meanwhile, due to the current lack of in vivo data, it remains difficult to establish clear nutritional recommendations for hypolipidemic functional foods. With further accumulation and improvement of experimental evidence in the future, appropriate dosages and application protocols will be gradually defined. TGase-mediated glycosylation offers outstanding advantages such as mild reaction conditions and high food safety, demonstrating good application potential in the food industry. However, its industrial economic feasibility, cost-effectiveness, and large-scale production potential compared to traditional modification methods still require comprehensive evaluation in future industry-oriented studies. Glycosylation modification has been proven to effectively improve sensory quality and mask the bitterness of marine protein hydrolysates. The sensory characteristics, flavor profile, and bitterness-masking effect of GUF3 are crucial for its practical food application, and related content will be systematically explored in our subsequent studies. Additionally, the specific glycosylation sites and precise structure-activity relationships of GUF3 will be the focus of our next research direction.

## CRediT authorship contribution statement

**Qianqian Huang:** Writing – original draft, Visualization, Validation, Software, Methodology, Data curation, Conceptualization. **Zhongqin Chen:** Supervision, Software, Investigation, Conceptualization. **Mingtang Tan:** Supervision, Software, Resources, Investigation. **Huina Zheng:** Validation, Methodology, Data curation. **Haisheng Lin:** Supervision, Software, Investigation. **Jialong Gao:** Supervision, Investigation. **Xiaoming Qin:** Project administration, Methodology. **Wenhong Cao:** Writing – review & editing, Supervision, Project administration, Funding acquisition.

## Declaration of competing interest

The authors declare that they have no known competing financial interests or personal relationships that could have appeared to influence the work reported in this paper.

## Data Availability

Data will be made available on request.

## References

[bb0005] Chang Y., Jiao Y., Li D.J., Liu X.L., Han H. (2022). Glycosylated zein as a novel nanodelivery vehicle for lutein. Food Chemistry.

[bb0010] Chen W.J., Ma X.B., Wang W.J., Lv R.L., Guo M.M., Ding T., Liu D.H. (2019). Preparation of modified whey protein isolate with gum acacia by ultrasound maillard reaction. Food Hydrocolloids.

[bb0015] Dong X., Cai Y.C., Liao H., Wang Y., Chen Z.H., Zhou Y., Xia X.L. (2024). Biological transformation of medicine and food homology hawthorn with Monascus ruber to enhance lipid-lowering function. Food Bioscience.

[bb0020] Du R., Li W., Li J.W., Zeng S., Chen Z.Q., Gao J.L., Cao W.H. (2024). Dynamic changes of zinc chemical speciation and zinc-containing peptides release in oysters (*Crassostrea hongkongensis*) during enzymatic hydrolysis. Food Bioscience.

[bb0025] Fukudome H., Yamaguchi T., Higuchi J., Ogawa A., Taguchi Y., Li J., Kabuki T., Ito K., Sakai F. (2021). Large-scale preparation and glycan characterization of sialylglycopeptide from bovine milk glycomacropeptide and its bifidogenic properties. Journal of Dairy Science.

[bb0030] Geng F., Xie Y.X., Wang Y., Wang J.Q. (2021). Depolymerization of chicken egg yolk granules induced by high-intensity ultrasound. Food Chemistry.

[bb0035] Gottardi D., Hong P.K., Ndagijimana M., Betti M. (2014). Conjugation of gluten hydrolysates with glucosamine at mild temperatures enhances antioxidant and antimicrobial properties. Lwt-Food Science and Technology.

[bb0040] Hong P.K., Gottardi D., Ndagijimana M., Betti M. (2014). Glycation and transglutaminase mediated glycosylation of fish gelatin peptides with glucosamine enhance bioactivity. Food Chemistry.

[bb0045] Huang Q.Q., Cao L., Chen Z.Q., Tan M.T., Zheng H.N., Lin H.S., Cao W.H. (2025). Cryoprotective effects and structural characteristics of collagen glycopeptides on. Lwt-Food Science and Technology.

[bb0050] Huang Q.Q., Song C.Y., Chen Z.Q., Tan M.T., Zheng H.N., Lin H.S., Cao W.H. (2025). Food-derived glycopeptides: Structural insights, glycosylation-driven bioactivity, and translational potential in health and functional applications. Trends in Food Science & Technology.

[bb0055] Jiang W., Wang C.Q., Zhai S.Y., Zhu W.T., Li J.X. (2024). Structural and functional properties of lactoferrin modified with carboxymethyl chitosan: Physical mixing and transglutaminase glycosylation. International Journal of Biological Macromolecules.

[bb0060] Lai W.W., Zhou S.P., Bai Y., Che Q.S., Cao H., Guo J., Su Z.Q. (2024). Glucosamine attenuates alcohol-induced acute liver injury via inhibiting oxidative stress and inflammation. Current Research in Food Science.

[bb0065] Li F., Zhang Z., Bai Y., Che Q., Cao H., Guo J., Su Z. (2023). Glucosamine improves non-alcoholic fatty liver disease induced by high-fat and high-sugar diet through regulating intestinal barrier function, liver inflammation, and lipid metabolism. Molecules.

[bb0070] Li Z.H., Jiang H., Guo M., Zhang Z., You X.Y., Wang X.P., Wang C.F. (2024). Effect of various oligosaccharides on casein solubility and other functional properties: Via Maillard reaction. International Journal of Biological Macromolecules.

[bb0075] Liu X.L., Wang J.T., Liu Y., Cui N., Wang D.Y., Zheng X.Q. (2022). Conjugation of the glutelin hydrolysates-glucosamine by transglutaminase and functional properties and antioxidant activity of the products. Food Chemistry.

[bb0080] Liu X.T., Yu Y.Q., Liu J.B., Wang D.F., Li S.R., Lyu S., Yang Q. (2025). Transglutaminase-catalyzed glycosylation of egg white peptides: Structural modulation and molecular mechanism of umami enhancement via T1R1/ T1R3 interactions. Food Research International.

[bb0085] Long G.H., Ji Y., Pan H.B., Sun Z.W., Li Y.T., Qin G.X. (2015). Characterization of thermal denaturation structure and morphology of soy glycinin by FTIR and SEM. International Journal of Food Properties.

[bb0090] Ma Y.Y., Xu J.J., Jiang S.S., Zeng M.Y. (2022). Effect of chitosan coating on the properties of nanoliposomes loaded with oyster protein hydrolysates: Stability during spray-drying and freeze-drying. Food Chemistry.

[bb0095] Meng Y., Zhao X., Jiang Y.Q., Ban Q.F., Wang X.B. (2023). Effect of Maillard reaction conditions on the gelation and thermal stability of whey protein isolate/D-tagatose conjugates. Food Chemistry.

[bb0100] Mirzaei M., Mirdamadi S., Safavi M., Soleymanzadeh N. (2020). The stability of antioxidant and ACE-inhibitory peptides as influenced by peptide sequences. Lwt-Food Science and Technology.

[bb0105] Moradi S.V., Hussein W.M., Varamini P., Simerska P., Toth I. (2016). Glycosylation, an effective synthetic strategy to improve the bioavailability of therapeutic peptides. Chemical Science.

[bb0110] Mudgil P., Baba W.N., Kamal H., FitzGerald R.J., Hassan H.M., Ayoub M.A., Maqsood S. (2022). A comparative investigation into novel cholesterol esterase and pancreatic lipase inhibitory peptides from cow and camel casein hydrolysates generated upon enzymatic hydrolysis and in-vitro digestion. Food Chemistry.

[bb0115] Mudgil P., Kamal H., Yuen G.C., Maqsood S. (2018). Characterization and identification of novel antidiabetic and anti-obesity peptides from camel milk protein hydrolysates. Food Chemistry.

[bb0120] Parandi E., Mousavi M., Assadpour E., Kiani H., Jafari S.M. (2024). Sesame protein hydrolysate-gum Arabic Maillard conjugates for loading natural anthocyanins: Characterization, in vitro gastrointestinal digestion and storage stability. Food Hydrocolloids.

[bb0125] Park J., Lee Y., Jung E.H., Kim S.M., Cho H., Han I.O. (2020). Glucosamine regulates hepatic lipid accumulation by sensing glucose levels or feeding states of normal and excess. Biochimica et Biophysica Acta-Molecular and Cell Biology of Lipids.

[bb0130] Qian J.J., Zheng L., Zhao Y.J., Zhao M.M. (2022). Stability, bioavailability, and structure-activity relationship of casein-derived peptide YPVEPF with a sleep-enhancing effect. Journal of Agricultural and Food Chemistry.

[bb0135] Qu W., Zhang X., Han X., Wang Z., He R., Ma H. (2018). Structure and functional characteristics of rapeseed protein isolate-dextran conjugates. Food Hydrocolloids.

[bb0140] Ryu T., Chang Y., Yoo J.J., Lee S.H., Jeong S.W., Kim S.G., Jang J.Y. (2025). Glucosamine supplementation attenuates progression of metabolic dysfunction-associated steatotic liver disease and related comorbidities. Clinical Nutrition.

[bb0145] Shang J.G., Zhong F., Zhu S., Wang J.L., Huang D.J., Li Y. (2020). Structure and physiochemical characteristics of whey protein isolate conjugated with xylose through Maillard reaction at different degrees. Arabian Journal of Chemistry.

[bb0150] Shen H.Y., Wang J., Ao J.F., Ye L.X., Shi Y.B., Liu Y.J., Luo A.W. (2023). The inhibitory mechanism of pentacyclic triterpenoid acids on pancreatic lipase and cholesterol esterase. Food Bioscience.

[bb0155] Sheng Y.Y., Yang W.L., Li Y.X., Xia W.X., Fu X.Y. (2025). Preparation of ginsenoside re-ginseng protein nanoparticles and their ex vivo and in vivo studies. Food Bioscience.

[bb0160] Song, Y., Li, J., & Wang, R. (2016). Structure and solubility of gluten-polysaccharide conjugates. Journal of the Chinese Cereals and Oils Association, 31(12), 125-131+138. doi:10.3969/j.issn.1003-0174.2016.12.023.

[bb0165] Tang P.L., Koh X.J. (2023). Ultrasound-assisted enzymatic hydrolysis enhances anti-inflammatory and hypoglycemic activities of edible bird’s nest. Food Bioscience.

[bb0170] Wang H.M., Jiang Y.J., Shi J. (2025). Effect of ultrasound combined with TGase-type glycation on the structure, physicochemical, and functional properties of casein hydrolysate. Ultrasonics Sonochemistry.

[bb0175] Wang K., Li W.C., Wang K., Hu Z.Y., Xiao H., Du B., Zhao L. (2022). Structural and inflammatory characteristics of Maillard reaction products from litchi thaumatin-like protein and fructose. Food Chemistry.

[bb0180] Wang L.Z., Jiang Z.M., Tian B., Bai L.N., Shi X.H., Zhang X.N. (2016). Effects of galactose concentration on characteristics of angiotensin-I-converting enzyme inhibitory peptides derived from bovine casein in Maillard reaction. International Journal of Food Properties.

[bb0185] Wang X.J., Liu X.L., Zheng X.Q., Qu Y., Shi Y.G. (2020). Preparation of corn glycopeptides and evaluation of their antagonistic effects on alcohol-induced liver injury in rats. Journal of Functional Foods.

[bb0190] Wang Y., Jiang Y.J., Shi J. (2024). Fabrication of novel casein/oligochitosan nanocomplexes for lutein delivery: Enhanced stability, bioavailability, and antioxidant properties. Food Research International.

[bb0195] Wu Y.T., Lu Y.Y., Huang Y.H., Lin H., Chen G.Z., Chen Y., Li Z.X. (2022). Glycosylation reduces the allergenicity of turbot *(Scophthalmus maximus)* parvalbumin by regulating digestibility, cellular mediators release and Th1/Th2 immunobalance. Food Chemistry.

[bb0200] Xu J.J., Jiang S.S., Liu L., Zhao Y.H., Zeng M.Y. (2021). Encapsulation of oyster protein hydrolysates in nanoliposomes: Vesicle characteristics, storage stability, release, and gastrointestinal digestion. Journal of Food Science.

[bb0205] Yang P., Li X., Wang H., Liu G. (2023). Analysis of the effect of different reducing sugars on Ara h2 glycation based on spectral technology. Spectroscopy and Spectral Analysis.

[bb0210] Yang R., Zuo P., Zhang M., Meng D.M., Wang B.W., Zhen T.Y. (2019). Transglutaminase induced oligochitosan glycosylation of ferritin as a novel nanocarrier for food bioactive molecules. Food Hydrocolloids.

[bb0215] Yang S., Zhang G., Chu H., Du P., Li A., Liu L., Li C. (2023). Changes in the functional properties of casein conjugates prepared by Maillard reaction with pectin or arabinogalactan. Food Research International.

[bb0220] Yu X., Liu R., Xu Y., Pan N., Liu X., Nakumura Y., Zhou D. (2025). Mechanism of TGase-induced oyster peptide-chitosan oligosaccharide glycosylation and amelioration effects of its zinc complex on zinc deficiency-associated neuron damages and oxidative stress. Food Research International.

[bb0225] Zeng Q.H., Hu D., Li Y.F., Qi P., Chen L., Wang R.H., Wang J.J. (2024). Conjugates of glycosylated antarctic krill proteins and curcumin: Maintaining the storage quality of salmon fillets coupling with photodynamic inactivation. Food Hydrocolloids.

[bb0230] Zhang A.Q., Cui Q., Yu Z.C., Wang X.B., Zhao X.H. (2021). Effects of transglutaminase glycosylated soy protein isolate on its structure and interfacial properties. Journal of the Science of Food and Agriculture.

[bb0235] Zhang X.Y., Wang Y., Li Z.Y., Li Y., Qi B.K. (2024). Effects of polysaccharide type on the structure, interface behavior, and foam properties of soybean protein isolate hydrolysate-polysaccharide Maillard conjugates. Food Hydrocolloids.

[bb0240] Zhang Y., Li C., Geary T., Jardim A., He S.D., Simpson B.K. (2022). Cold setting of gelatin-antioxidant peptides composite hydrogels using a new psychrophilic recombinant transglutaminase (rTGase). Food Hydrocolloids.

[bb0245] Zhao C., Jiang Y.J., Shi J. (2026). Underlying the effect of TGase-catalyzed glycosylation of fish gelatin: Perspective on structural characteristics, physico-chemical properties and functional properties. Food Hydrocolloids.

[bb0250] Zhao M.G., He H., Ma A.M., Hou T. (2023). Sources, chemical synthesis, functional improvement and applications of food-derived protein/peptide-saccharide covalent conjugates: A review. Critical Reviews in Food Science and Nutrition.

[bb0255] Zhao Z.L., Wang W.D., Chen J., Chen J.X., Deng J.Y., Wu G.X., Luo D.H. (2024). Effect of ultrasound-assisted Maillard reaction on functional properties and flavor characteristics of oyster protein enzymatic hydrolysates. Ultrasonics Sonochemistry.

[bb0260] Zheng Y.M., Chang Y., Luo B.Y., Teng H., Chen L. (2022). Molecular structure modification of ovalbumin through controlled glycosylation with dextran for its emulsibility improvement. International Journal of Biological Macromolecules.

[bb0265] Zhu G.P., Zhang C.H., Qin X.M., Cao W.H., Zheng H.N., Gao J.L. (2021). Ameliorative effects of oyster protein hydrolysate on age-induced cognitive impairment via restoring glia cell dysfunction and neuronal injured in zebrafish. Journal of Functional Foods.

[bb0270] Zhu Q.Y., Xu W.H., Zhang C.Q., Gong J.B., Qin X.G., Zhang H.Z., Liu G. (2024). Transglutaminase-mediated glycosylation enhances the physicochemical and functional properties of ovalbumin. Food Hydrocolloids.

