# Supplementary material for: Transglutaminase-mediated glycosylation improves the physicochemical properties and in vitro hypolipidemic activity of oyster peptides
Source: Food Chem X. 2026 May 23;36:104027. doi: 10.1016/j.fochx.2026.104027 (PMC13226887; doi:10.1016/j.fochx.2026.104027)
Supplement: Supplementary material [file mmc1.docx]

**
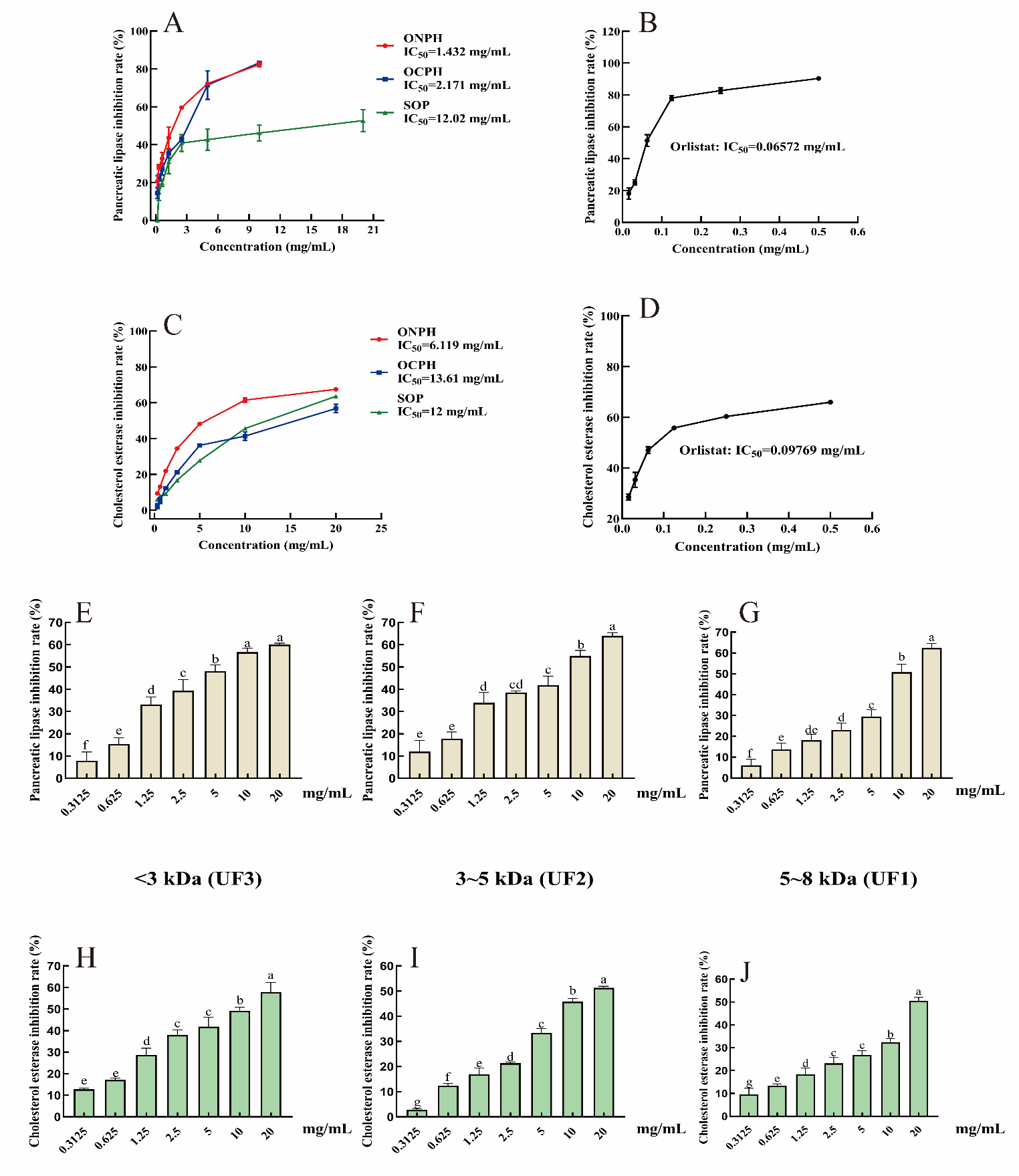
 Figure S1.** Inhibitory effects of oyster hydrolysate and its ultrafiltration fractions on pancreatic lipase and cholesterol esterase. Data are presented as mean ± standard deviation. Different lowercase letters indicate statistically significant differences (*p* < 0.05).


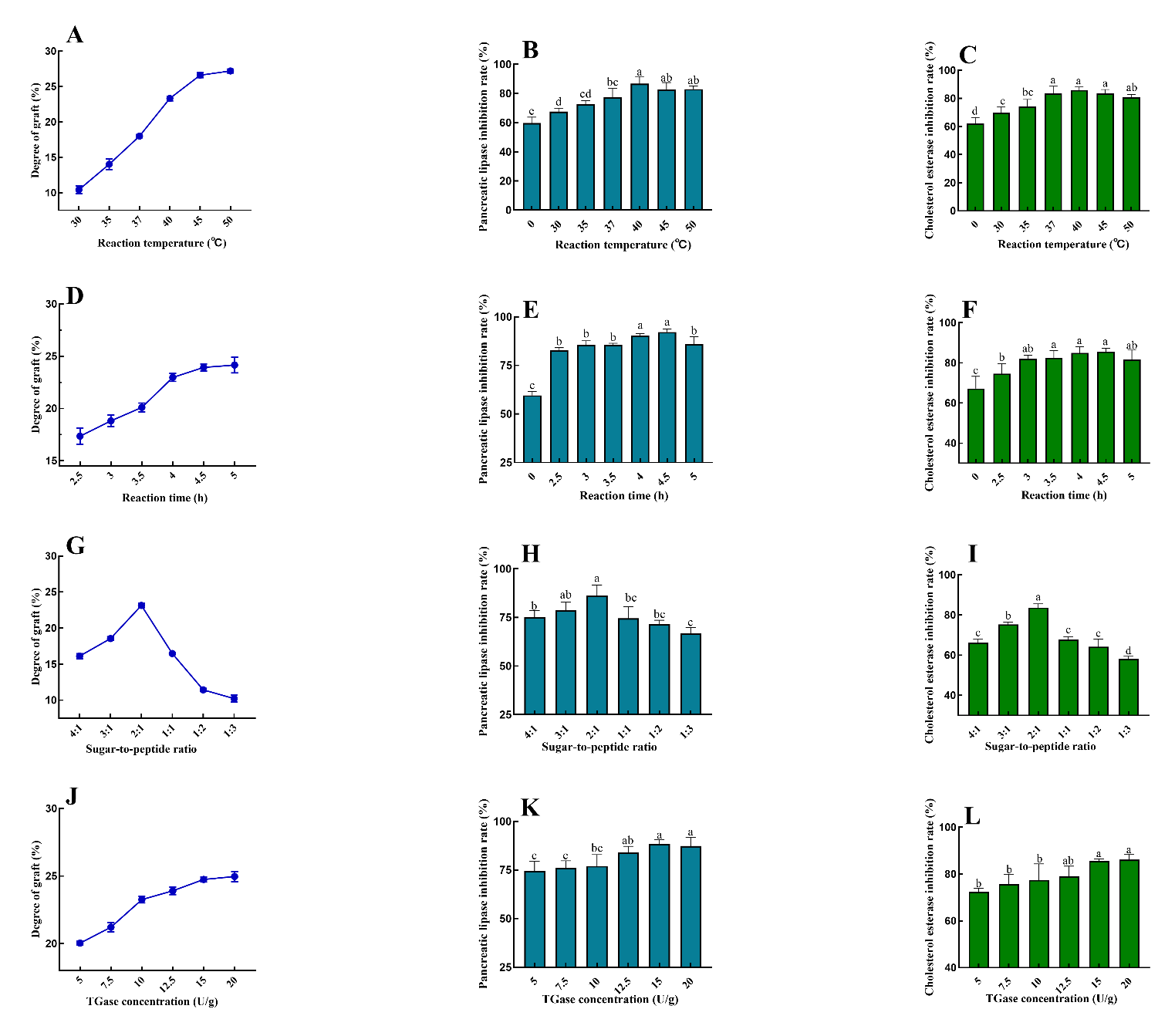


**Figure S2.** Effects of different reaction conditions on the degree of grafting of glycosylation products and their inhibitory activities against pancreatic lipase and cholesterol esterase. Data are presented as mean ± standard deviation.
